# Human-Origin Influenza A(H3N2) Reassortant Viruses in Swine, Southeast Mexico

**DOI:** 10.3201/eid2504.180779

**Published:** 2019-04

**Authors:** Martha I. Nelson, Carine K. Souza, Nídia S. Trovão, Andres Diaz, Ignacio Mena, Albert Rovira, Amy L. Vincent, Montserrat Torremorell, Douglas Marthaler, Marie R. Culhane

**Affiliations:** National Institutes of Health, Bethesda, Maryland, USA (M.I. Nelson, N.S. Trovão);; National Animal Disease Center, Ames, Iowa, USA (C.K. Souza, A.L. Vincent);; Icahn School of Medicine at Mount Sinai, New York, New York, USA (N.S. Trovão, I. Mena);; University of Minnesota, Saint Paul, Minnesota, USA (A. Diaz, A. Rovira, M. Torremorell, D. Marthaler, M.R. Culhane)

**Keywords:** influenza A virus, influenza virus, reassortant influenza viruses, viruses, influenza, swine, evolution, phylogenetic analysis, antigenic cartography, reassortment, phylogeography, pandemic, human origin, reverse zoonosis, zoonoses, vaccine, respiratory infections, Mexico

## Abstract

The genetic diversity of influenza A viruses circulating in swine in Mexico complicates control efforts in animals and presents a threat to humans, as shown by influenza A(H1N1)pdm09 virus. To describe evolution of swine influenza A viruses in Mexico and evaluate strains for vaccine development, we sequenced the genomes of 59 viruses and performed antigenic cartography on strains from 5 regions. We found that genetic and antigenic diversity were particularly high in southeast Mexico because of repeated introductions of viruses from humans and swine in other regions in Mexico. We identified novel reassortant H3N2 viruses with genome segments derived from 2 different viruses that were independently introduced from humans into swine: pandemic H1N1 viruses and seasonal H3N2 viruses. The Mexico swine viruses are antigenically distinct from US swine lineages. Protection against these viruses is unlikely to be afforded by US virus vaccines and would require development of new vaccines specifically targeting these diverse strains.

Genetically diverse influenza A viruses (IAV) circulate in swine globally, complicating efforts to control the virus and increasing the threat that a novel virus will emerge in pigs with the capacity to infect humans. This threat was exemplified by the influenza A(H1N1)pdm09 virus, which originated in swine in Mexico, most likely in the west or central–north regions (*1*). Although IAVs have been endemic in US swine herds since 1919 (*2*), there is no evidence that IAVs circulated in swine in Mexico before the 1990s. The emergence of Mexico as a hub of swine IAV diversity with pandemic potential is a relatively recent event.

During 1989–2015, >2 million hogs raised in the United States were transported to Mexico, representing >87% of all US swine exports (Figure 1, panel A). During the 1990s, ≈100,000 live hogs were transported annually, on average, from the United States to Mexico (Figure 1, panel B), thus facilitating establishment of 2 major North American swine IAV lineages in Mexico by the end of the decade: triple reassortant swine H3N2 (TRswH3N2) viruses and classical swine H1N1 (CswH1N1) viruses (*1*) (referred to as lineage 1A according to recently proposed H1 nomenclature [*3*]). Avian-like Eurasian swine H1N1 (EAswH1N1, lineage 1C [*3*]) viruses were also introduced into multiple regions of Mexico from Europe. Previously, EAswH1N1 viruses had only been detected in Europe and Asia (*4*–*6*), and Mexico is the only country in the Americas where TRswH3N2, CswH1N1, and EAswH1N1 lineages co-circulate and exchange genome segments by reassortment (*1*).

**Figure 1:**
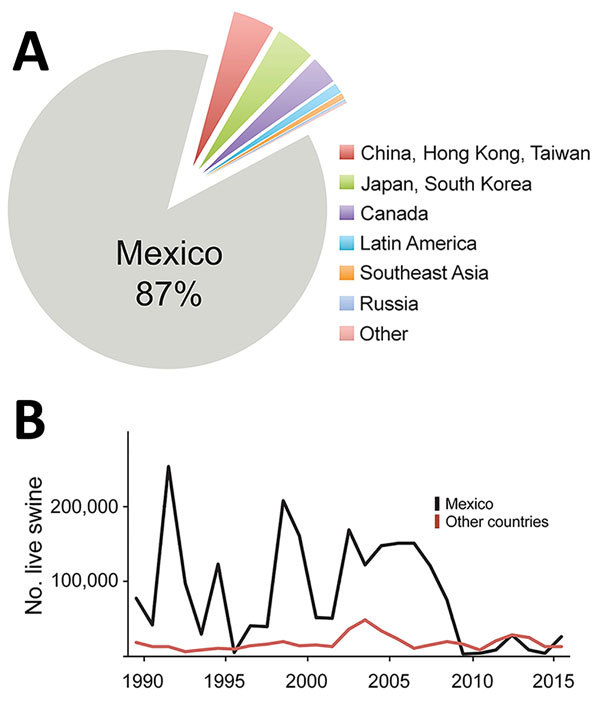
Swine exportation to Mexico from the United States and eradication of CSFV in Mexico. A) Hogs exported from the United States to other countries globally during 1989–2015. Of ≈3.7 million exported, ≈3.1 million (≈87%) were exported to Mexico. B) Since 1989, the number of hogs exported from the United States to Mexico has experienced year-to-year variation. Data are available from the US International Trade Commission (https://dataweb.usitc.gov).

Increased surveillance of swine IAV in Mexico and other understudied countries has elucidated how international trade of live swine drives the long-distance migration of these viruses. For many decades, countries with less-established swine production have imported breeding stock from North America and Europe, facilitating long-range dissemination of swine IAV lineages to China and Southeast Asia (*4*,*7*,*8*). Swine movements between US regions regularly facilitate long-range swine IAV migration across the country (*9*), but long-distance movements of pigs between regions in Mexico are less frequent.

The 6 largest swine-producing states in Mexico are located in the west (Jalisco, ≈2.8 million swine), northwest (Sonora, ≈1.7 million swine), east (Puebla, ≈1.6 million swine and Veracruz, ≈1.5 million swine), central–north (Guanajuato, ≈0.9 million swine), and southeast (Yucatan, ≈0.9 million swine) regions (Figure 2). During 1973–2009, Mexico implemented a campaign to eradicate classical swine fever virus (CSFV), including restrictions on animal movements between CSFV-free areas and central regions experiencing outbreaks (*10*) (Figure 2). Lifting of internal movement controls during 2009 provided new opportunities for swine IAV to migrate between regions in Mexico.

**Figure 2:**
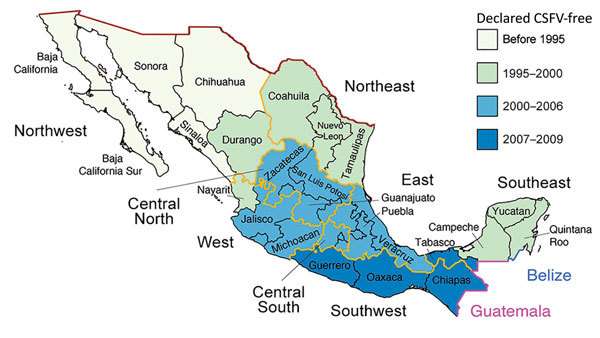
Swine exportation to Mexico from the United States and eradication of CSFV in Mexico. The 32 states of Mexico shaded according to the year when CSFV was declared to be eradicated by the Secretariat of Agriculture, Livestock, Rural Development, Fishery and Food of the government of Mexico. International borders are shaded between Mexico and the United States (red), Belize (blue), and Guatemala (violet). The 8 regions of Mexico are indicated, and their borders are shaded orange. CSFV, classical swine fever virus.

To determine the genetic and antigenic diversity of swine IAVs circulating in Mexico, we sequenced genomes of 59 swine IAV samples collected in northwest and southeast Mexico (GenBank accession nos. MG836712–831) (Appendix Table 1). We also evaluated the relationship of Mexico swine IAVs to those used in existing vaccines to determine whether new vaccine development is necessary.

## Materials and Methods

### Collection of Swine IAV in Mexico

Respiratory tract samples were collected by veterinarians and farm staff from pigs with clinical signs of respiratory disease on farms in the northwest and southeast regions of Mexico during 2010–2014. These samples were sent ad hoc to the University of Minnesota Veterinary Diagnostic Laboratory (St. Paul, MN, USA) for swine respiratory disease diagnostic investigations. This laboratory conducted matrix gene testing for swine IAV by using a real-time reverse transcription PCR (RT-PCR) (*11*) and virus isolation in MDCK cells (*12*) for swine IAV PCR-positive samples, as requested by the submitting party. Hemagglutinin (HA) and neuraminidase (NA) subtyping of RT-PCR–positive samples was completed (*13*). Sequencing of the HA, but not other segments, was performed previously (*14*).

We obtained whole-genome sequences from virus isolates or directly from the originally submitted respiratory tract material. RNA was extracted from the original material or virus isolates as described (*14*). In brief, we extracted virus RNA from 50 μL of swab supernatant by using a magnetic bead procedure and obtained segment-specific PCR fragments by using the One-Step RT-PCR (QIAGEN, https://www.qiagen.com) and IAV-specific primers for each genome segment as described (*15*). We obtained 59 complete genomes, 37 from southeast Mexico and 22 from northwest Mexico. All sequences were submitted to GenBank under accessions nos. MG836712–831.

### Phylogenetic Analysis

We generated nucleotide alignments for each of the 8 segments of the virus genome (HA, matrix protein [MP], NA, nucleoprotein [NP], nonstructural [NS], polymerase acidic [PA], polymerase basic 1 [PB1], and polymerase basic 2 [PB2]) by using MUSCLE version 3.8.31 (*16*). We inferred initial trees for each segment by using neighbor-joining methods to identify major lineages, including pandemic (p), TRswH3N2 (t), CswH1N1 (c), and EAswH1N1 (e): PB2t, PB1p, PB1t, PAp, PAt, H1c, H1p, H3t + H3h, NPp, NPt, N1c, N1e, N1p, N2t + N2h, MPe, MPp, MPt, NSp, and NSt. For simplicity, we visualized human H3N2 (huH3N2) and TRswH3N2 together on the H3 and N2 phylogenies. As additional background data, we downloaded related human and swine sequences from the Influenza Virus Resource (*17*) that were studied previously, including swine viruses from Mexico, which are indicated with the abbreviation AVX (e.g., A/swine/Mexico/AVX-24/2012[H1N1]) (*1*).

For each pandemic H1N1 virus alignment, we inferred the phylogenetic relationships by using the maximum-likelihood method in RAxML version 7.2.6 (*18*) and incorporated a general time-reversible model of nucleotide substitution with a gamma-distributed rate variation among sites. To assess the robustness of each node, we performed a bootstrap resampling process (500 replicates). We used the high-performance computational capabilities of the Biowulf Linux cluster at the National Institutes of Health (Bethesda, MD, USA) (http://biowulf.nih.gov). For TRswH3N2, EAswH1N1, CswH1N1, and huH3N2, we inferred time-scaled phylogenies by using Markov chain Monte Carlo (MCMC) methods available in the BEAST package version 1.8.4 (*19*) and used a relaxed uncorrelated lognormal molecular clock, a constant population demographic model, and a general time-reversible model of nucleotide substitution with a gamma-distributed rate variation among sites. We ran the MCMC chain separately 3 times for each of the datasets for >100 million iterations, with subsampling every 10,000 iterations.

We used the BEAGLE library (*20*) to improve computational performance. All parameters reached convergence, as assessed visually by using Tracer version 1.6 in BEAST; statistical uncertainty was reflected by values of the 95% highest posterior density. The initial 10% of the chain was removed as burn-in, runs were combined by using LogCombiner version 1.8.4 in BEAST, and maximum clade credibility trees were summarized by using TreeAnnotator version 1.8.4 in in BEAST. We classified H1 viruses by using the H1 swine clade classification tool available at the Influenza Research Database (http://www.fludb.org) (*3*) (Appendix Table 1).

We inferred the timing of the introduction of human seasonal H3N2 viruses into swine in southeast Mexico on the basis of the time to the most recent common ancestor of the clade of viruses identified in swine and the most closely related human viruses, sampled in the mid-1990s. Inferring the precise timing of human-to-swine transmission events that occurred many decades ago is complicated by the lack of sequence data available from swine in previous decades, and the most parsimonious interpretation is that long branch lengths indicate gaps in sampling in swine, rather than humans, an approach that has been described in detail (*13*).

### Spatial Analysis

The 31 states of Mexico (plus the Federal District) are located within 8 defined regions (Figure 2). We conducted phylogeographic analysis at a regional level to ensure deidentification for swine producers. Swine IAV sequences were available for 5 regions in Mexico (northwest, west, central–north, east, and southeast). To reduce complexity for maximum clade credibility, we categorized viruses categorized as northwest region (NW), southeast region (SE), or central-north (CN), east (E), or west (W) regions.

We categorized global background sequences as USA/Canada, Asia, Europe, South America, and humans (globally). The location state was specified for each virus sequence, which enabled the expected number of location state transitions in the ancestral history conditional on the data observed at the 3 tips to be estimated by using Markov jump counts (*21*), providing a quantitative measure of asymmetry in gene flow between regions. The location of viruses in the pandemic H1N1 clade (pH1N1) was left uninformed, which enabled the reconstruction of the location state of the common ancestor of pH1N1 to be unbiased by human data. For computational efficiency we conducted the phylogeographic analysis by using an empirical distribution of 1,000 trees (*22*), running the MCMC chain for 25 million iterations, and sampling every 1,000 steps. We used a nonreversible diffusion model and Bayesian stochastic search variable selection to improve statistical efficiency for all datasets containing >4 location states. Heat maps were constructed by using the R package to summarize Markov jump counts inferred over phylogenies for all segments and swine IAV lineages (*23*).

### Movements of Live Swine

We obtained the number of US live swine exports during 1989–2015 from the US International Trade Commission (https://dataweb.usitc.gov/scripts/REPORT.asp) by using HS Code 0103. Data were cross-referenced against the United Nations Commodity Trade Statistics Database (http://comtrade.un.org), which provides the trade value (in US dollars) for live swine trade between countries for the years 1996–2012. We obtained the estimated live swine population size of Mexico from the Mexico Secretariat of Agriculture, Livestock, Rural Development, Fisheries and Food (https://www.siap.gob.mx). Information on movements of live swine between Mexican states is available only for the relatively small number of pigs that go through plants that pass official government inspections, known as Tipo Inspección Federal plants.

Information on the year of CSFV eradication and opening of border restrictions in each state of Mexico was based on reports of the Secretariat of Agriculture, Livestock, Rural Development, Fisheries and Food. We corroborated these data with reports from the Animal and Plant Health Inspection Service of the US Department of Agriculture (US National Archives) (https://www.federalregister.gov; document nos. 02-11897, 02-24753, and E7-10641).

### Antigenic Characterization

Representative H3N2 strains from the United States and Mexico and a human seasonal strain (Appendix Table 2) genetically related to the unique H3N2 swine virus introduced into Mexico were selected from sequence and motif analyses and used for hemagglutination inhibition (HI) assays. The analysis included 5 swine influenza viruses from Mexico reported by Mena et al. (*1*). Monovalent antiserum against swine IAV H3N2 strains was prepared in swine as described (*24*) and used for HI assays (*25*) with 5 H3N2 swine viruses from Mexico as antigens. HI data were used to determine the antigenic relationships between swine H3 from Mexico and the United States by using antigenic cartography 3-dimensional maps (*24*). Antigenic distances between viruses were calculated in antigenic units (AUs), in which 1 AU is equivalent to a 2-fold loss in HI cross-reactivity (Appendix Table 3). Antigenic distances generated in the 3-dimensional map between the H3N2 antigens were plotted by using GraphPad Prism Version 7.03 (https://www.graphpad.com).

## Results

### Genetic Diversity of Swine IAV in Mexico

**Figure 3:**
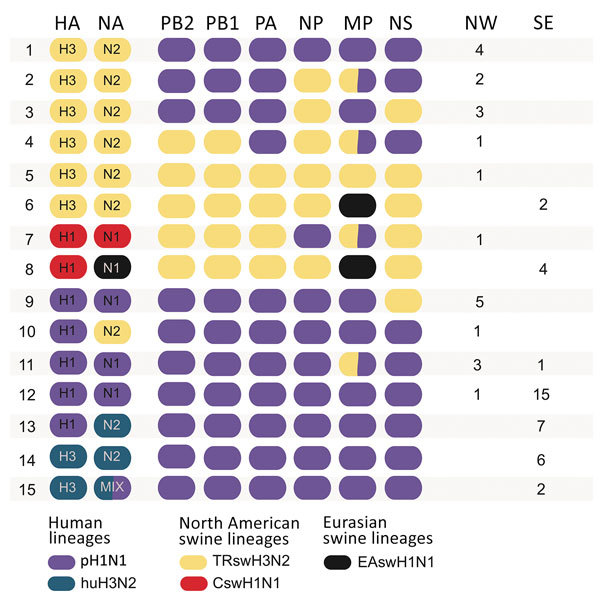
Genetic diversity of influenza A viruses (IAVs) circulating in swine in southeast and northwest Mexico. Fifteen genotypes were identified by surveillance in swine herds in Mexico during 2010–2014. Each oval represents 1 of the 8 segments of the virus genome. The surface antigens HA and NA are listed first, followed by the 6 internal gene segments. Shading of each oval corresponds to 1 of 5 major genetic lineages of swine IAV circulating in humans and swine globally. The rightmost columns indicate the number of viruses from each genotype collected from the northwest and southeast regions in Mexico. Ovals shaded with 2 colors represent mixed infections. CswH1, classical swine H1N1; EAswH1, avian-like Eurasian swine H1N1; HA, hemagglutinin; huH3N2, human H3N2; MP, matrix protein; NA, neuraminidase; NP, nucleoprotein; NS, nonstructural; NW, northwest; PA, polymerase acidic; PB, polymerase basic; pH1N1, pandemic H1N1 clade; SE, southeast; SW, southwest.TRswH3N2, triple reassortant swine H3N2.

Phylogenetic analysis of the 59 swine IAV whole-genome sequences generated for this study showed that extensive genetic diversity is circulating in the northwest and southeast regions of Mexico. This viral diversity was produced by 3 evolutionary processes: human-to-swine transmission, long-distance movements of swine, and genomic reassortment. We identified 15 genotypes, including 13 reassortant genotypes with segments from multiple IAV lineages (Figure 3). Three IAV lineages were observed in swine in northwest Mexico: human-origin pH1N1 (1A.3.3.2), TRswH3, and CswH1. These 3 lineages also were identified in southeast Mexico, along with 2 additional lineages: EAswH1N1, likely introduced from pigs in Europe, and huH3N2, which appears to have been introduced from humans into swine in the 1990s. Eight reassortant genotypes were identified that were not identified in swine in Mexico in previous studies (*1*), including 1 triple reassortant (TRswH3N2/pH1N1/EAswH1N1, genotype 6) (Figure 3).

Seven of the 8 reassortant genotypes identified in northwest Mexico were generated by reassortment between TRswH3N2 and pH1N1 viruses. Genotype 8 is similar to the pH1N1 precursor viruses identified previously in the west and central-north regions of Mexico (*1*) and represents the first detection of the genotype outside of the west and central-north regions (Figure 3). Approximately 35% (13/37) of the viruses identified in southeast Mexico were pH1N1/huH3N2 reassortants (Figure 3), in which internal genes were replaced with internal genes of a pH1N1 virus. Seven viruses had possible evidence of co-infection, but only in the MP segment. However, this finding requires further confirmation.

### Genetic Diversity of HA and NA Segments

**Figure 4:**
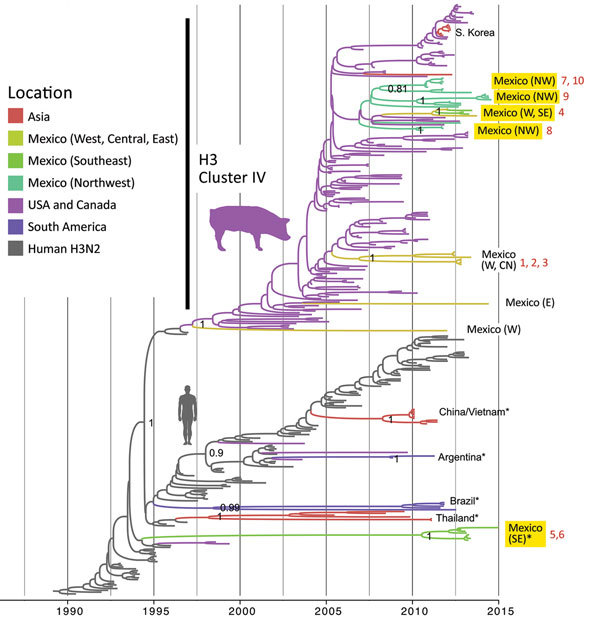
Evolutionary relationships between H3 segments of avian influenza viruses collected in humans and swine globally. Time-scaled Bayesian maximum clade credibility tree is inferred for the H3 segment. The tree includes newly generated sequences from northwest and southeast Mexico, along with background sequences from swine in Mexico, Asia, the United States, and Canada, as well as seasonal H3N2 viruses from humans. The color of each branch indicates the most probable location state. Posterior probabilities for key nodes are provided. Clades with viruses from swine in Mexico obtained for this study are highlighted in yellow. *Major virus introductions into a location, indicating direct introductions from humans. CN, central; NW, northwest; S, south; SE, southeast; W, west. The 10 H3N2 viruses selected for antigenic characterization (Figure 6) are indicated by numbers 1–10 in red. A more detailed phylogeny, including tip labels and all posterior probabilities, is provided in the Appendix Figure.

**Figure 5:**
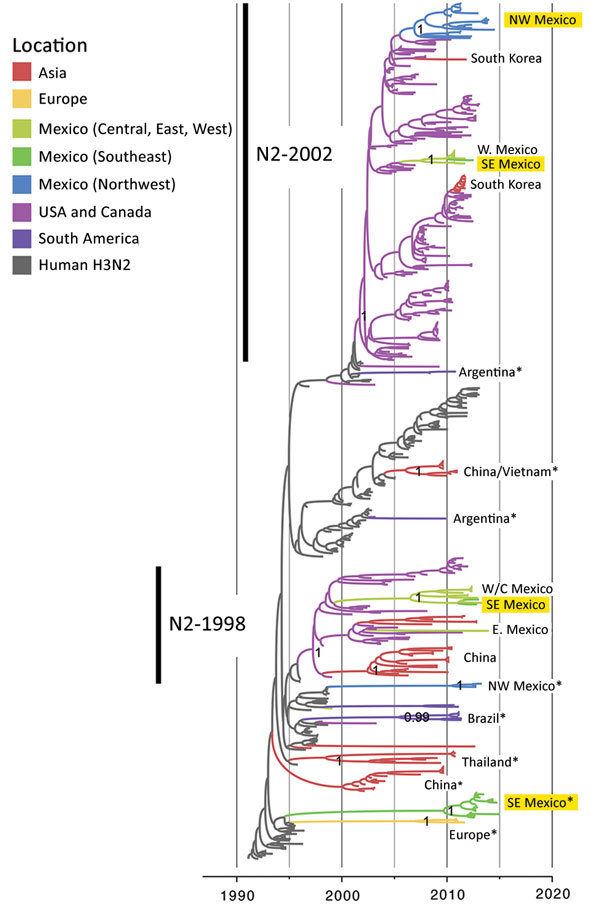
Evolutionary relationships between N2 segments of avian influenza viruses collected in humans and swine globally. Time-scaled Bayesian maximum clade credibility tree is inferred for N2 segment. Labeling and shading is similar to that in Figure 4, with the additional labeling of the N2–1998 and N2–2002 lineages. NW, northwest; SE, southeast; W, west; W/C, west/central. *Direct introduction from humans. A more detailed phylogeny, including tip labels and all posterior probabilities, is provided in the Appendix Figure.

Overall, greater genetic diversity was observed in the HA and NA antigens than in the 6 internal gene segments, a pattern that has been observed in other countries (*26*). CswH1N1-H1β (1A.2), pH1N1 (1A.3.3.2), and TRswH3N2 (cluster IV) viruses were identified in northwest Mexico. Five HAs (CswH1N1-H1γ [1A.3.3.3], pH1N1 [1A.3.3.2], TRswH3N2 [cluster IV], and huH3N2) (Figures 3, 4; Appendix Table 1) were identified in southeast Mexico. Five NA lineages (EAswH1N1, pH1N1, huH3N2, TRswH3N2/N2–1998, and TRswH3N2/N2–2002) (Figure 5) were identified in southeast Mexico. Given the high genetic diversity of H3 viruses, including huH3N2 viruses that were not phylogenetically related to any known US swine lineages (Figure 4), we selected 10 representative H3 viruses from Mexico on the basis of phylogenetic position (Figure 4) and antigenic motif patterns (*27*) for antigenic characterization.

### Antigenic Analysis of H3 Viruses

**Figure 6:**
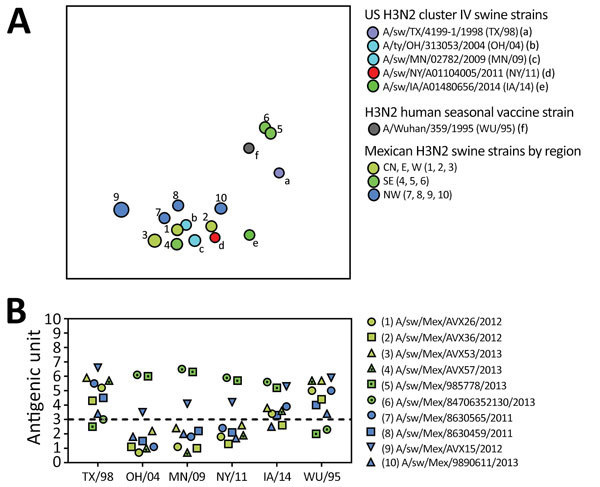
Antigenic relationships between contemporary influenza A (H3N2) viruses from Mexico and representative strains from the United States. A) Antigenic map of contemporary swine H3N2 viruses from Mexico and the United States. Antigenic clusters are indicated by color as in Figures 4 and 5. CN, central; E, east; NW, northwest; SE, southeast; W, west. B) Antigenic distance between H3N2 virus from Mexico and representative swine strains from the United States and a putative ancestral human seasonal vaccine virus strain (WU/95). The US swine strains represent antigenic clusters previously defined as TX/98 (cornflower), OH/04 and MN/09 (cyan), NY/11 (red), and IA/14 (green) (*27*,*31*).

We generated an antigenic map from HI data to visualize antigenic relationships among contemporary H3N2 swine IAV from multiple regions in Mexico and the United States (Figure 6, panel A). Antigenic distances were extracted from the map to measure distance between H3N2 viruses from Mexico and swine strains from the United States that represent putative antigenic clusters contained in US commercial vaccines (Figure 6, panel B). Of the 10 viruses from Mexico selected for antigenic characterization, 8 were antigenically clustered with H3N2 cluster IV strains from the United States. Two viruses from the southeast region (A/sw/Mex/985778/2013 and A/sw/Mex/84706352130/2015) were more antigenically similar to the human seasonal vaccine strain WU/95 (<3 AUs). The most similar swine strain is the cluster I TX/98.

### Spatial Structure of Swine IAV in Mexico

**Figure 7:**
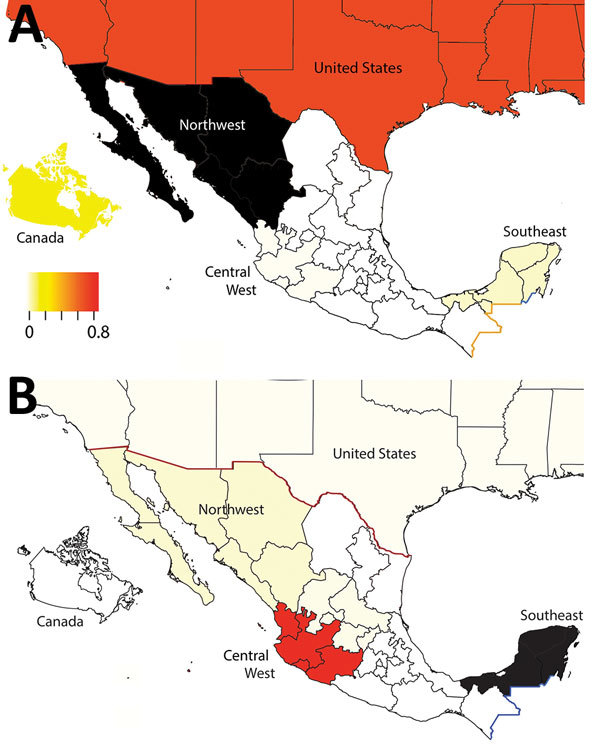
Sources of influenza A viruses circulating in swine in northwestern and southeastern Mexico. Each region is shaded according to the proportion of total Markov jump counts from that particular region (source) into A) northwest or B) southeast regions of Mexico (destination). Red indicates high proportion of jumps (major source of viruses); light yellow indicates low proportion of jumps (not a major source of viruses); black indicates destination; white indicates no jumps/no data available. Seven locations were considered in the analysis: Canada, United States, Mexico (northwest), Mexico (central-west), Mexico (central-north), Mexico (east), and Mexico (southeast). Scale bar indicates proportion of total Markov jump counts from a particular region.

We observed no evidence of viral migration between the northwest and southeast regions of Mexico. Although certain clades of pH1N1 viruses from northwest and southeast Mexico share a common ancestor, the low posterior probabilities supporting these nodes indicate that multiple independent human-to-swine transmission events of closely related human pH1N1 viruses are more likely. There might have been 4 independent introductions of pH1N1 virus from humans into swine in southeast Mexico and 5 introductions into the northwest region (Appendix Figure). Similarly, there is no evidence of dispersal of huH3N2 viruses from southeast Mexico to any other regions of Mexico. TRswH3N2 and CswH1N1 viruses from swine in the United States were imported into the northwest region of Mexico, which is located near the US border (Figure 7, panel A), reflecting the large number of live swine transported from the southern United States into Mexico each year (Figure 1). In contrast, TRswH3N2 and CswH1N1 viruses were imported into the southeast region of Mexico primarily from the west region (Figure 7, panel B), an inference that was observed with high support across all phylogenies (Figures 4, 5; Appendix Figure).

**Figure 8:**
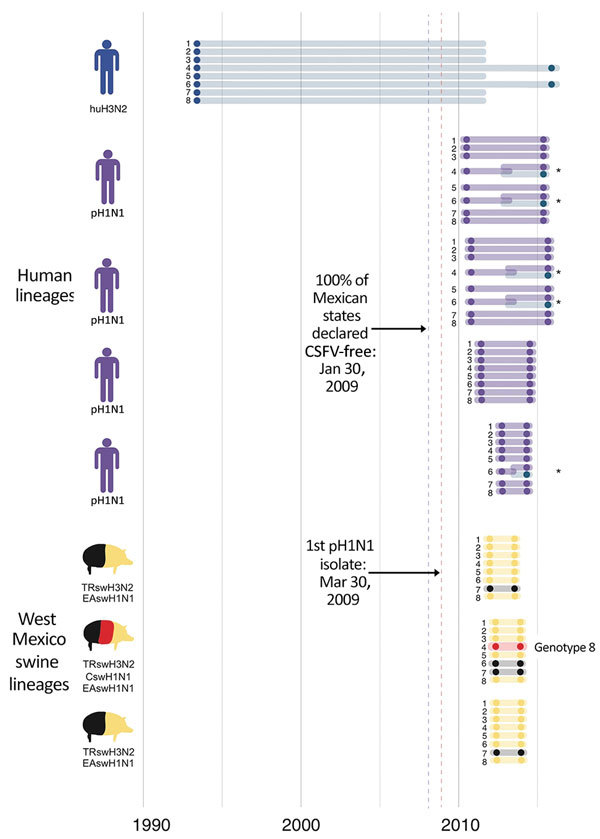
Introductions of influenza A viruses into swine in southeast Mexico. Introductions of these viruses from humans and swine into southeast Mexico are indicated as groups of 8 horizontal lines, and each line represents 1 of 8 segments of the virus genome. Segments are numbered 1–8, longest to shortest, according to convention: 1, polymerase basic 2; 2, polymerase basic 1; 3, polymerase acidic; 4, hemagglutinin; 5, nucleoprotein; 6, neuraminidase; 7, matrix protein; 8, nonstructural. The position of each shaded circle along the x-axis represents the estimated timing of virus introduction, estimated directly from the maximum clade credibility trees. Lines and circles are shaded according to the virus lineage, similar to that in Figure 2. *Reassortment events. Introduction of genotype 8 viruses is indicated. The blue dotted vertical line represents the date of the declaration of Secretariat of Agriculture, Livestock, Rural Development, Fisheries and Food in Mexico that classical swine fever virus had been eradicated from all states in Mexico during January 2009. The red dotted vertical line represents the date of first isolation of pH1N1 virus in humans. CSFV, classical swine fever virus; CswH1, classical swine H1N1; EAswH1, avian-like Eurasian swine H1N1; huH3N2, human H3N2; pH1N1, pandemic H1N1 clade; TRswH3N2, triple reassortant swine H3N2.

The trees indicate that 3 independent introductions of reassortant viruses, including EAswH1N1 segments (genotypes 6 and 8) (Figure 3), occurred during ≈2011–2013 on the basis of estimated times to the most recent common ancestor (Figure 8; Appendix Table 4). No additional reassortment events have been observed between genotype 6 or 8 viruses and huH3N2 and pH1N1 viruses also circulating in southeast Mexico.

## Discussion

The recent characterization of the origins of the influenza A(H1N1)pdm09 virus in swine in Mexico underscored the need for efforts to control the unexpectedly diverse swine IAV populations circulating in large swine herds in this country (*1*). Long-range animal movements between Europe, Mexico, and the United States established an exceptionally diverse population of swine IAV in the west and central-north regions of Mexico, including EAswH1N1 reassortants, which have now disseminated onward into southeast Mexico. Recognition of the genetic diversity of swine IAV in Mexico, including lineages not found in US herds, has stimulated interest from producers in Mexico in developing new vaccines customized to protect against the virus strains in this country, including huH3N2 viruses in southeast Mexico that were introduced from humans in the 1990s and are highly divergent from all known US swine virus strains. The huH3N2 viruses have recently reassorted with pH1N1 viruses, generating novel reassortant genotypes that were identified frequently in southeast Mexico. Eighty percent of H3N2 viruses in swine in southeast Mexico had the HA from the huH3N2 lineage. Our analysis indicates that the huH3N2 viruses have been circulating in swine for many decades, consistent with being fit, well-adapted viruses in pigs. However, the breadth of our sampling is insufficient to draw strong conclusions about whether these viruses are widespread in southeast Mexico, and further surveillance is critical.

Producers invest considerable time and resources into efforts to control swine IAV through vaccination, particularly of sows (*28*). Influenza vaccines are licensed for swine in Europe and North America, including multivalent formulations targeting the genetically diverse virus populations found in these regions (*29*,*30*). Mexico uses some vaccines made in North America, but relies heavily on customized (autogenous) vaccines designed from strains isolated in the field. US producers also frequently use autogenous vaccines because commercial vaccine formulations cannot keep up with the rapid emergence of antigenically novel swine IAV lineages. The H3N2 antigenic maps demonstrate that swine IAV strains detected in most parts of Mexico were antigenically more similar to older US cluster IV strains that circulated during 2004–2011 (*31*). Two swine IAV strains from the southeast region of Mexico retained more cross-reactivity with the Wuhan/95 human seasonal H3N2 vaccine strain, compared with other human virus strains and US cluster IV swine viruses, supporting the phylogenetic inference of a separate human-to-swine introduction in the 1990s in Mexico. Although these 2 strains also demonstrated antigenic relatedness to US cluster I swine virus TX/98, phylogenetic analysis indicates that the introduction into Mexico was separate from that into the United States, albeit from an antigenically similar human seasonal H3N2 ancestor. With such long branch lengths on the phylogenetic tree, we cannot exclude the possibility that human-to-swine transmission occurred in a different country where surveillance of swine is low and where movements of pigs into Mexico could have introduced the virus (e.g., Guatemala), but at this time there is no evidence for such a scenario.

**Figure 9:**
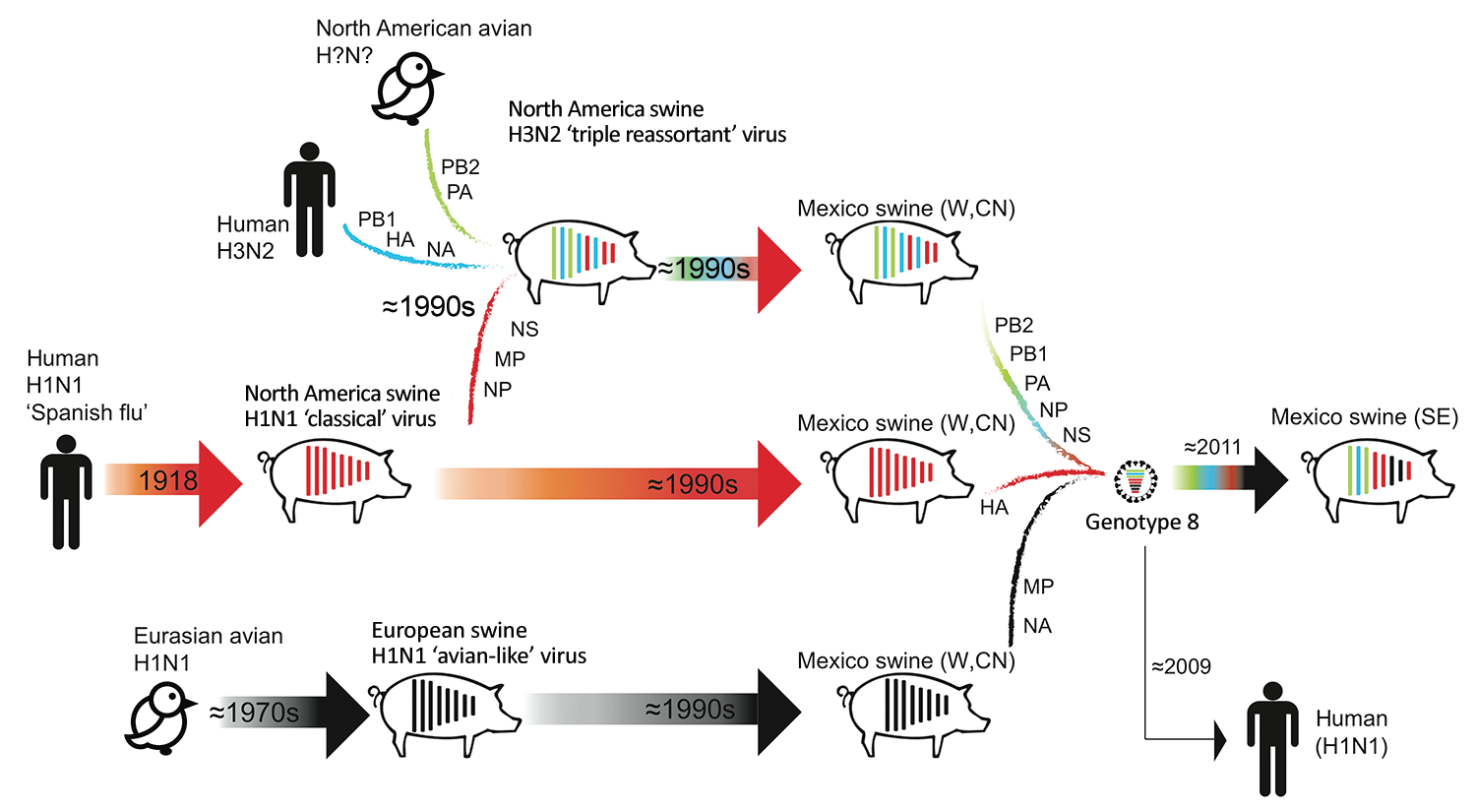
Evolutionary history of genotype 8 influenza viruses in southeast Mexico, showing interspecies transmission events, reassortment events, and virus migration events leading to the emergence of genotype 8 viruses in swine in Mexico in the W and CN regions and continued spread into SE Mexico in ≈2011. Within each pig, vertical lines represent the 8 segments of the influenza A virus genome, ordered longest to shortest, which are shaded according to virus lineage. Arrows represent direct transmission events between hosts. Reassortment events are indicated by multiple lines shaded in different colors. Segments donated from each lineage are indicated. Birds are depicted to reflect uncertainty about the specific avian species that transmitted influenza A viruses to swine in Europe and North America. CN, central; E, east; HA, hemagglutinin; MP, matrix protein; NA, neuraminidase, NP, nucleoprotein; NS, nonstructural; PA, polymerase acidic; PB, polymerase basic; SE, southeast; W, west.

Increased surveillance in swine in the years after the 2009 H1N1 pandemic (*32*) has enhanced our understanding of swine IAV evolution. Whereas movements of humans and wild birds tend to follow symmetric routes of workflows (*33*), flight patterns (*22*), and flyways (*34*), the movements of pigs are often asymmetric, inconsistent with standard gravity models, and driven by fluctuating economic considerations. We demonstrated how genetic diversity that evolved in regions of central Mexico has spread >1,000 miles to southeast Mexico and introduced new lineages, including those of Eurasian origin. Introduction of genotype 8 viruses into southeast Mexico is notable, given their role in the genesis of the 2009 H1N1 pandemic in humans and their complex evolutionary history involving multiple intercontinental migration events and interspecies transmission events (Figure 9). Time-scaled phylogenies consistently indicate that the west region of Mexico is the original source of the relatively recent (≈2011–2013) incursions of reassortant viruses into the southeast region. Removal of movement restrictions in 2009 might have facilitated virus flow between these regions because the final 3 states to eradicate CSFV in 2009 (Chiapas, Oaxaca, and Tabasco) are geographically situated between the southeast and west/central-north regions of Mexico (Figure 2). However, as is the case in most countries, there are no reliable data on pig movements within Mexico, and our phylogeographic methods cannot exclude the possibility of virus movement through unsampled intermediary locations.

Trade volumes of live swine from the United States into Mexico can fluctuate by orders of magnitude (Figure 1, panel B), driven by economic factors and disease-control efforts, such as during the outbreak of porcine reproductive and respiratory syndrome virus in the United States (*35*). However, it is difficult to explore temporal links with international virus movements given that no swine IAV sequence data for Mexico are available before 2009 (*36*). The increase in swine IAV surveillance in Mexico since 2009 has been critical for evaluating potential vaccine effectiveness and catalyzing development of new vaccine formulations. Earlier detection of newly emergent strains and better predictions about whether new strains will increase in prevalence will require deeper, population-based surveillance of IAV in swine. Given limited public funding available for surveillance, advances in vaccine effectiveness will require a culture of greater transparency among producers, and guarantees that data shared will not be used to target individual producers or countries.

Appendix Additional information on human-origin influenza A(H3N2) reassortant viruses in swine, southeast Mexico.
