# Supplementary material for: Human-Origin Influenza A(H3N2) Reassortant Viruses in Swine, Southeast Mexico
Source: Emerg Infect Dis. 2019 Apr;25(4):691–700. doi: 10.3201/eid2504.180779 (PMC6433011; doi:10.3201/eid2504.180779)
Supplement: Appendix — Additional information on human-origin influenza A(H3N2) reassortant viruses in swine, southeast Mexico. [file 18-0779-Techapp-s1.pdf]

Article DOI: https://doi.org/10.3201/eid2504.180779

Human-Origin Influenza A(H3N2) Reassortant Viruses in Swine, Southeast Mexico

Appendix

Appendix Table 1. Characteristics of 59 influenza viruses sequenced for this analysis, southeast Mexico*

Virus name	Date of isolation	Location in Mexico	PB2	PB1	PA	HA	H1	NP	NA	MP	NS	Genotype
A/Swine/Mexico/9684432/2013/Mixed	2013 Feb 25	SE	P	P	P	H3 (human)		P	mixed	P	P	15
A/Swine/Mexico/9783514/2013/Mixed	2013 Apr 15	SE	P	P	P	H3 (human)		P	mixed	P	P	15
A/Swine/Mexico/9557133/2013/H1N1	2013 Jan 2	SE	T	T	T	H1c (gamma)	1A.3.1	T	N1e	MP-e	T	8
A/Swine/Mexico/9762101/2013/H1N1	2013 Apr 6	SE	T	T	T	H1c (gamma)	1A.3.1	T	N1e	MP-e	T	8
A/Swine/Mexico/9783445/2013/H1N1	2013 Apr 15	SE	T	T	T	H1c (gamma)	1A.3.1	T	N1e	MP-e	T	8
A/Swine/Mexico/9800323/2013/H1N1	2013 Apr 22	SE	T	T	T	H1c (gamma)	1A.3.1	T	N1e	MP-e	T	8
A/Swine/Mexico/9453312/2012/H3N2	2012 Oct 29	SE	P	P	P	H3 (human)		P	N2 (human)	P	P	14
A/Swine/Mexico/9634750/2013/H3N2	2103 Jan 31	SE	P	P	P	H3 (human)		P	N2 (human)	P	P	14
A/Swine/Mexico/9714185/2013/H3N2	2013 Mar 14	SE	P	P	P	H3 (human)		P	N2 (human)	P	P	14
A/Swine/Mexico/9741876/2013/H3N2	2013 Mar 28	SE	P	P	P	H3 (human)		P	N2 (human)	P	P	14
A/Swine/Mexico/9857788/2013/H3N2	2013 May 20	SE	P	P	P	H3 (human)		P	N2 (human)	P	P	14
A/Swine/Mexico/84706352130/2015/H3N2	2015 Jun 9	SE	P	P	P	H3 (human)		P	N2 (human)	P	P	14
A/Swine/Mexico/10032432/2013/H1N2	2013 Aug 16	SE	P	P	P	P	1A.3.3.2	P	N2 (human)	P	P	13
A/Swine/Mexico/10290795/2014/H1N2	2013 Dec 22	SE	P	P	P	P	1A.3.3.2	P	N2 (human)	P	P	13
A/Swine/Mexico/10912920/2014/H1N1	2014 Nov 5	SE	P	P	P	P	1A.3.3.2	P	N2 (human)	P	P	13
A/Swine/Mexico/10926242/2014/H1N2	2014 Nov 12	SE	P	P	P	P	1A.3.3.2	P	N2 (human)	P	P	13
A/Swine/Mexico/84703289210/2015/H1N2	2015 Mar 25	SE	P	P	P	P	1A.3.3.2	P	N2 (human)	P	P	13
A/Swine/Mexico/9711817/2013/H1N2	2103 Mar 11	SE	P	P	P	P	1A.3.3.2	P	N2 (human)	P	P	13
A/Swine/Mexico/9800379/2013/H1N2	2013 Apr 22	SE	P	P	P	P	1A.3.3.2	P	N2 (human)	P	P	13
A/Swine/Mexico/9562931/2013/H3N2	2102 Dec 23	SE	T	T	T	H3 (swine)		T	N2 (swine)	MP-e	T	6
A/Swine/Mexico/9783350/2013/H3N2	2013 Apr 15	SE	T	T	T	H3 (swine)		T	N2 (swine)	MP-e	T	6
A/Swine/Mexico/9199572/2012/H1N1	2102 Jun 28	SE	P	P	P	P	1A.3.3.2	P	P	Mixed	P	11
A/Swine/Mexico/9922474/2013/H1N1	2013 Jun 22	SE	P	P	P	P	1A.3.3.2	P	P	P	P	12
A/Swine/Mexico/10126158/2013/H1N1	2013 Sep 30	SE	P	P	P	P	1A.3.3.2	P	P	P	P	12
A/Swine/Mexico/10248405/2013/H1N1	2013 Dec 1	SE	P	P	P	P	1A.3.3.2	P	P	P	P	12
A/Swine/Mexico/10433030/2014/H1N1	2014 Mar 1	SE	P	P	P	P	1A.3.3.2	P	P	P	P	12
A/Swine/Mexico/10466772/2014/H1N1	2014 Mar 16	SE	P	P	P	P	1A.3.3.2	P	P	P	P	12
A/Swine/Mexico/84704755140/2014/H1N1	2014 Aug 28	SE	P	P	P	P	1A.3.3.2	P	P	P	P	12
A/Swine/Mexico/10969586/2014/H1N1	2014 Dec 4	SE	P	P	P	P	1A.3.3.2	P	P	P	P	12
A/Swine/Mexico/9213802/2012/H1N1	2012 Jul 5	SE	P	P	P	P	1A.3.3.2	P	P	P	P	12
A/Swine/Mexico/9425974/2012/H1N1	2012 Oct 14	SE	P	P	P	P	1A.3.3.2	P	P	P	P	12

Virus name	Date of isolation	Location in Mexico	PB2	PB1	PA	HA	H1	NP	NA	MP	NS	Genotype
A/Swine/Mexico/9713846/2013/H1N1	2013 Mar 14	SE	P	P	P	P	1A.3.3.2	P	P	P	P	12
A/Swine/Mexico/9722783/2013/H1N1	2013 Mar 17	SE	P	P	P	P	1A.3.3.2	P	P	P	P	12
A/Swine/Mexico/9741916/2013/H1N1	2013 Mar 28	SE	P	P	P	P	1A.3.3.2	P	P	P	P	12
A/Swine/Mexico/9800385/2013/H1N1	2013 Apr 22	SE	P	P	P	P	1A.3.3.2	P	P	P	P	12
A/Swine/Mexico/9800380/2013/H1N1	2013 Apr 22	SE	P	P	P	P	1A.3.3.2	P	P	P	P	12
A/Swine/Mexico/9800390/2013/H1N1	2013 Apr 22	SE	P	P	P	P	1A.3.3.2	P	P	P	P	12
A/Swine/Mexico/8935602/2012/H1N1	2012 Feb 23	NW	T	T	T	H1c (β)	1A.2	P	N1c	Mixed	T	7
A/Swine/Mexico/10499819/2014/H3N2	2014 Apr 3	NW	P	P	P	H3 (swine)		P	N2 (swine)	P	P	1
A/Swine/Mexico/847061253020/2014/H3N2	2014 Apr 28	NW	P	P	P	H3 (swine)		P	N2 (swine)	P	P	1
A/Swine/Mexico/847071253020/2014/H3N2	2014 Apr 28	NW	P	P	P	H3 (swine)		P	N2 (swine)	P	P	1
A/Swine/Mexico/10598944/2014/H3N2	2014 Jun 13	NW	P	P	P	H3 (swine)		P	N2 (swine)	P	P	1
A/Swine/Mexico/7932475/2010/H3N2	2010 Nov 4	NW	P	P	P	H3 (swine)		T	N2 (swine)	Mixed	T	2
A/Swine/Mexico/8630588/2011/H3N2	2011 Sep 26	NW	P	P	P	H3 (swine)		T	N2 (swine)	Mixed	T	2
A/Swine/Mexico/8630565/2011/H3N2	2011 Sep 26	NW	P	P	P	H3 (swine)		T	N2 (swine)	P	T	3
A/Swine/Mexico/8630638/2011/H3N2	2011 Sep 26	NW	P	P	P	H3 (swine)		T	N2 (swine)	P	T	3
A/Swine/Mexico/9890611/2013/H3N2	2013 Jun 6	NW	P	P	P	H3 (swine)		T	N2 (swine)	P	T	3
A/Swine/Mexico/8630459/2011/H3N2	2011 Sep 26	NW	T	T	P	H3 (swine)		T	N2 (swine)	Mixed	P	4
A/Swine/Mexico/7773583/2010/H3N2	2010 Aug 22	NW	T	T	T	H3 (swine)		T	N2 (swine)	MPc	T	5
A/Swine/Mexico/11004418/2014/H1N2	2014 Dec 22	NW	P	P	P	P	1A.3.3.2	P	N2 (swine)	P	P	10
A/Swine/Mexico/8630529/2011/H1N1	2011 Sep 26	NW	P	P	P	P	1A.3.3.2	P	P	P	T	9
A/Swine/Mexico/84703178820/2014/H1N1	2014 Jun 13	NW	P	P	P	P	1A.3.3.2	P	P	P	T	9
A/Swine/Mexico/84703857030/2014/H1N1	2014 Jun 25	NW	P	P	P	P	1A.3.3.2	P	P	P	T	9
A/Swine/Mexico/847011987050/2014/H1N1	2014 Oct 23	NW	P	P	P	P	1A.3.3.2	P	P	P	T	9
A/Swine/Mexico/847021987050/2014/H1N1	2014 Oct 23	NW	P	P	P	P	1A.3.3.2	P	P	P	T	9
A/Swine/Mexico/7774063/2010/H1N1	2010 Aug 22	NW	P	P	P	P	1A.3.3.2	P	P	Mixed	P	11
A/Swine/Mexico/7942103/2010/H1N1	2010 Nov 7	NW	P	P	P	P	1A.3.3.2	P	P	Mixed	P	11
A/Swine/Mexico/8630550/2011/H1N1	2011 Sep 26	NW	P	P	P	P	1A.3.3.2	P	P	Mixed	P	11
A/Swine/Mexico/9583196/2013/H1N1	2013 Jan 4	NW	P	P	P	P	1A.3.3.2	P	P	P	P	12

*Genotypes refer to those listed in Figure 2. HA, hemagglutinin; MP, matrix protein; NA, neuraminidase; NP, nucleoprotein; NS, nonstructural; NW, northwest; PA, polymerase acidic; PB, polymerase basic; SE, southeast; SW, southwest.

Appendix Table 2. Times to the most recent common ancestor for introduction of influenza viruses into southeast Mexico*

<table>
<tr><th></th><th colspan="4">Genotype</th></tr>
<tr><th>Characteristic</th><th>6</th><th>8</th><th>6</th><th>14 + 15</th></tr>
<tr><td>Viruses</td><td>A/sw/Mexico/9562931/2013/H3N2
A/sw/Mexico/9783350/2013/H3N2</td><td>A/sw/Mexico/9783445/2013/H1N1</td><td>A/sw/Mexico/AVX-57/2013/H3N2</td><td>A/Swine/Mexico/84706352130/2015/H3N2
A/Swine/Mexico/9453312/2012/H3N2
A/Swine/Mexico/9714185/2013/H3N2
A/Swine/Mexico/9741876/2013/H3N2
A/Swine/Mexico/9857788/2013/H3N2
A/Swine/Mexico/9634750/2013/H3N2
A/Swine/Mexico/9684432/2013/Mixed
A/Swine/Mexico/9783514/2013/Mixed</td></tr>
<tr><td></td><td></td><td colspan="2">A/sw/Mexico/9762101/2013/H1N1
A/sw/Mexico/9800323/2013/H1N1
A/sw/Mexico/9557133/2013/H1N1</td><td></td></tr>
<tr><td>Origin</td><td>Central Mexico</td><td>Central Mexico</td><td>Central Mexico</td><td>Humans</td></tr>
<tr><td>Destination</td><td>East Mexico</td><td>East Mexico</td><td>East Mexico</td><td>East Mexico</td></tr>
<tr><td>PB2-t</td><td>2011.1–2012.79</td><td>2012.5–2012.8</td><td></td><td></td></tr>
<tr><td>PB1-t</td><td>2012.22–2012.9</td><td>2011.06–2012.69</td><td></td><td></td></tr>
<tr><td>PA-t</td><td>2010.73–2012.64</td><td>2011.28–2012.26</td><td></td><td></td></tr>
<tr><td>H1c</td><td></td><td>2011.23–2012.76</td><td></td><td></td></tr>
<tr><td>H3</td><td colspan="2">2011.25–2012.85</td><td>2012.12–2013.47</td><td>1994.32–2010.55</td></tr>
<tr><td>NP-t</td><td>2011.48–2012.64</td><td>2012.06–2012.85</td><td colspan="2">2012.0–2013.47</td></tr>
<tr><td>N1-e</td><td></td><td colspan="2">2011.06–2012.71</td><td></td></tr>
<tr><td>N2</td><td colspan="2">2009.95–2012.98</td><td>2011.55–2013.47</td><td>1994.55–2009.7</td></tr>
<tr><td>MP-e</td><td>2011.85–2012.69</td><td>2012.35–2012.7</td><td colspan="2">2011.98–2013.47</td></tr>
<tr><td>NS-t</td><td>2011.29– 2012.42</td><td>2011.73–2012.46</td><td colspan="2">2011.76– 2013.47</td></tr>
<tr><td>Average</td><td>2011.23–2012.74</td><td>2011.66–2012.65</td><td>2011.92–2013.47</td><td>1994.43–2010.12</td></tr>
</table>

*H, hemagglutinin; MP, matrix protein; NA, neuraminidase; NP, nucleoprotein; NS, nonstructural; NW, northwest; PA, polymerase acidic; PB, polymerase basic.

Appendix Table 3. Influenza virus strains used in antigenic analysis of influenza virus, southeast Mexico*

GenBank accession no.	Strain	Country	Antigenic map strains reference
KM821302	A/Wuhan/359/1995†	China	Wu/95
CY095675	A/swine/TX/4199–2/1998	USA	TX/98
EU735818	A/turkey/OH/313053/2004	USA	OH/04
CY099103.1	A/swine/MN/02782/2009	USA	MN/09
JN940422.1	A/swine/NY/A01104005/2011	USA	NY/11
KJ635928.1	A/swine/IA/A01480656/2014	USA	IA/14
KU976701.1	A/swine/Mex/AVX26/2012	Mexico	1
KU976600.1	A/swine/Mex/AVX36/2012	Mexico	2
KU976665.1	A/swine/Mex/AVX53/2013	Mexico	3
KU976721.1	A/swine/Mex/AVX57/2013	Mexico	4
KR870290.1	A/swine/Mex/9857788/2013	Mexico	5
MG836811.1	A/swine/Mex/84706352130/2015	Mexico	6
KR870257.1	A/swine/Mex/8630565/2011	Mexico	7
KR870254.1	A/swine/Mex/8630459/2011	Mexico	8
KU976510.1	A/swine/Mex/AVX15/2012	Mexico	9
KR870291.1	A/swine/Mex/9890611/2013	Mexico	10

*Includes 5 strains isolated from swine in Mexico, as reported by Mena et al. (*1*) and indicated by the abbreviation AVX.
†Influenza A(H3N2) human seasonal vaccine virus strain.

Appendix Table 4. Hemagglutination titers between viruses from Mexico and reference strains from swine and humans in the United States*

Antigen	Virus type	A(H3N2) /Wuhan/ 359/1995	A(H3N2) /Wuhan/ 359/ 1995	A(H3N2) /swine/ Texas/ 4199–1/ 1998	A(H3N2 /swine/ Texas/ 4199–1/ 1998	A(H3N2) /turkey/ Ohio/ 313053/ 2004	A(H3N2) /turkey/ Ohio/ 313053/ 2004	A(H3N2)/ swine/ Minnesota/ 02782/ 2009	A(H3N2)/ swine/ Minnesota/ 02782/ 2009	A(H3N2)/ swine/ New York/ A011040 05/2011	A(H3N2)/ swine/ New York/ A011040 05/ 2011	A(H3N2) /swine /Iowa/ A014806 56/2014	A(H3N2) /swine/ Iowa/ A014806 56/2014
A(H3N2)/Wuhan/359/1995	Human vaccine	640	640	640	640	80	40	–	–	80	80	80	160
A(H3N2)/swine/Texas/4199–1/1998	Cluster I	160	320	2,560	1,280	40	20	320	320	20	40	40	80
A(H3N2)/turkey/Ohio/313053/ 2004	Cluster IV	10	20	40	20	640	640	–	–	320	160	20	20
A(H3N2)/swine/Minnesota/02782/ 2009	Cluster IV	<10	20	80	40	–	–	5,120	2,560	320	640	40	20
A(H3N2)/swine/New York/A01104005/2011	Cluster IV-A	20	20	320	160	160	80	1,280	1,280	640	1,280	160	160
A(H3N2)/swine/Iowa/A01480656/ 2014	Cluster IV-A	40	40	160	80	160	40	160	320	320	320	640	1,280
A(H3N2)/swine/Mexico/AVX26/ 2012		<10	10	80	20	320	320	–	–	640	640	40	20
A(H3N2)/swine/Mexico/AVX36/ 2012		10	40	160	80	1,280	640	–	–	320	320	40	20
A(H3N2)/swine/Mexico/AVX53/ 2012		<10	10	40	20	40	20	–	–	40	80	20	20
A(H3N2)/swine/Mexico/AVX57/ 2012		10	20	160	40	320	80	–	–	160	160	20	<10
A(H3N2)/swine/Mexico/985778/ 2013		40	80	80	80	–	–	40	40	20	20	<10	<10
A(H3N2)/swine/Mexico/ 84706352130/2015		20	80	80	80	–	–	40	20	10	20	<10	<10
A(H3N2)/swine/Mexico/8630565/ 2011		20	20	80	40	–	–	1,280	1,280	160	80	20	<10
A(H3N2)/swine/Mexico/8630459/ 2011		40	80	160	80	–	–	2,560	2,560	160	160	40	10
A(H3N2)/swine/Mexico/AVX15/ 2012		20	20	<10	10	40	20	–	–	320	160	<10	<10
A(H3N2)/swine/Mexico/9890611/ 2013		40	80	160	640	–	–	1,280	640	160	80	10	<10

*–, not tested.

Reference

1. Mena I, Nelson MI, Quezada-Monroy F, Dutta J, Cortes-Fernández R, Lara-Puente JH, et al. Origins of the 2009 H1N1 influenza pandemic in swine in Mexico. eLife. 2016;5:e16777. PubMed http://dx.doi.org/10.7554/eLife.16777

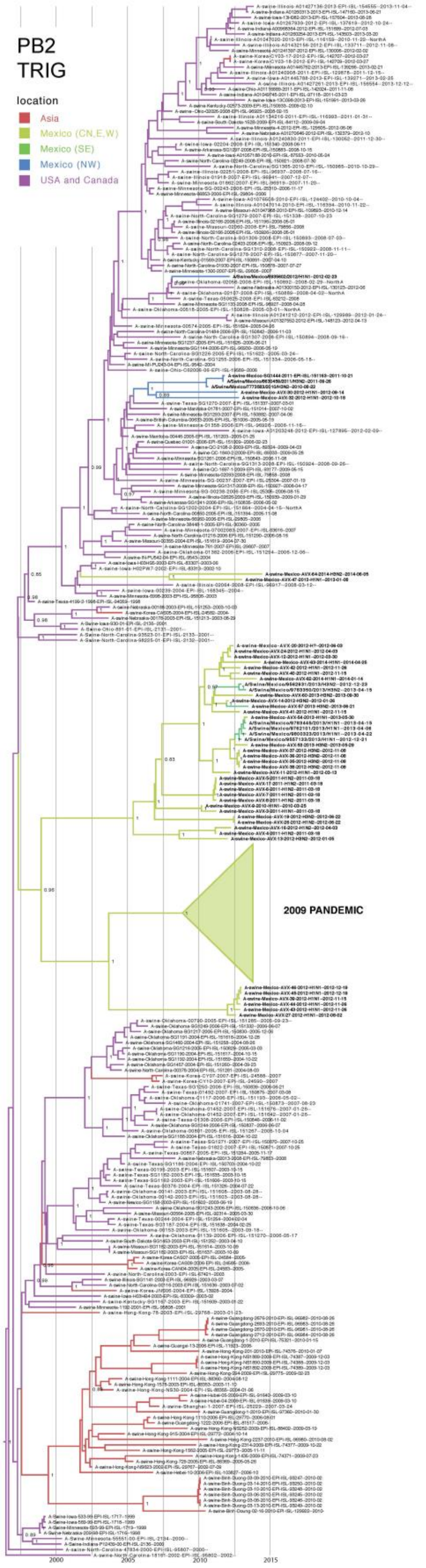
PB2
TRIG
location
Asia
Mexico (CN,E,W)
Mexico (SE)
Mexico (NW)
USA and Canada
2009 PANDEMIC
2000
2005
2010
2015

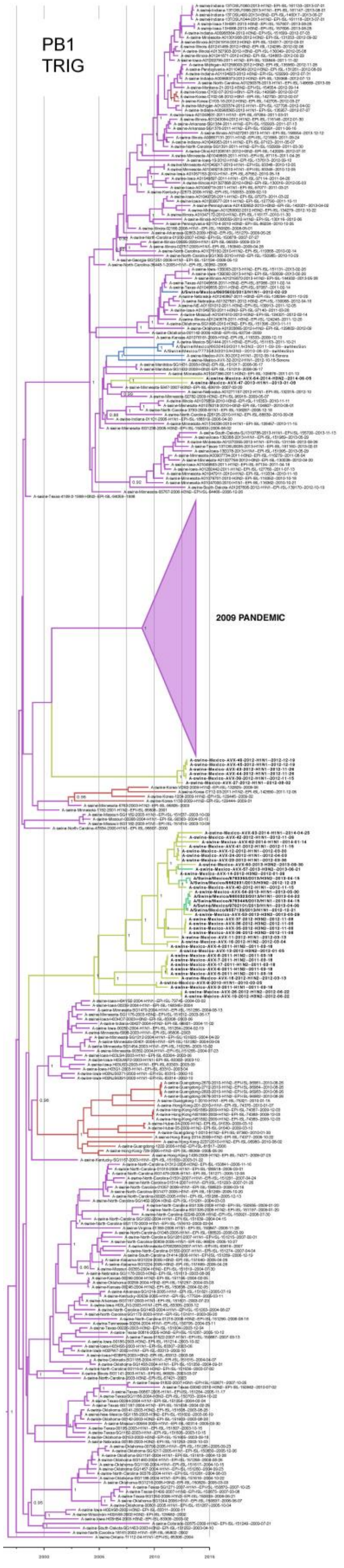
PB1
TRIG
2009 PANDEMIC

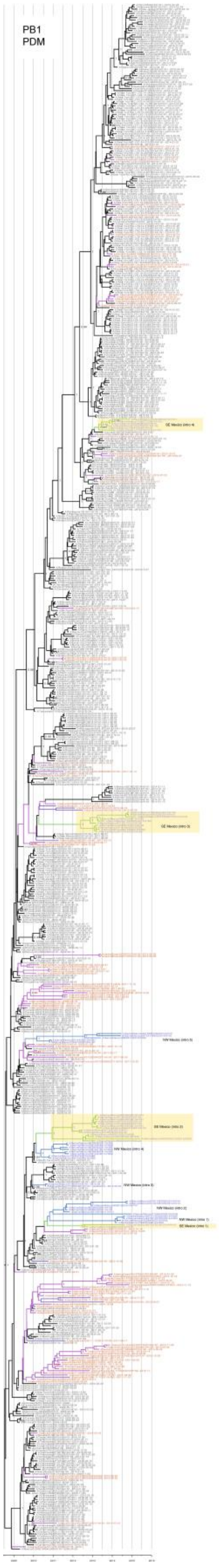
PB1
PDM

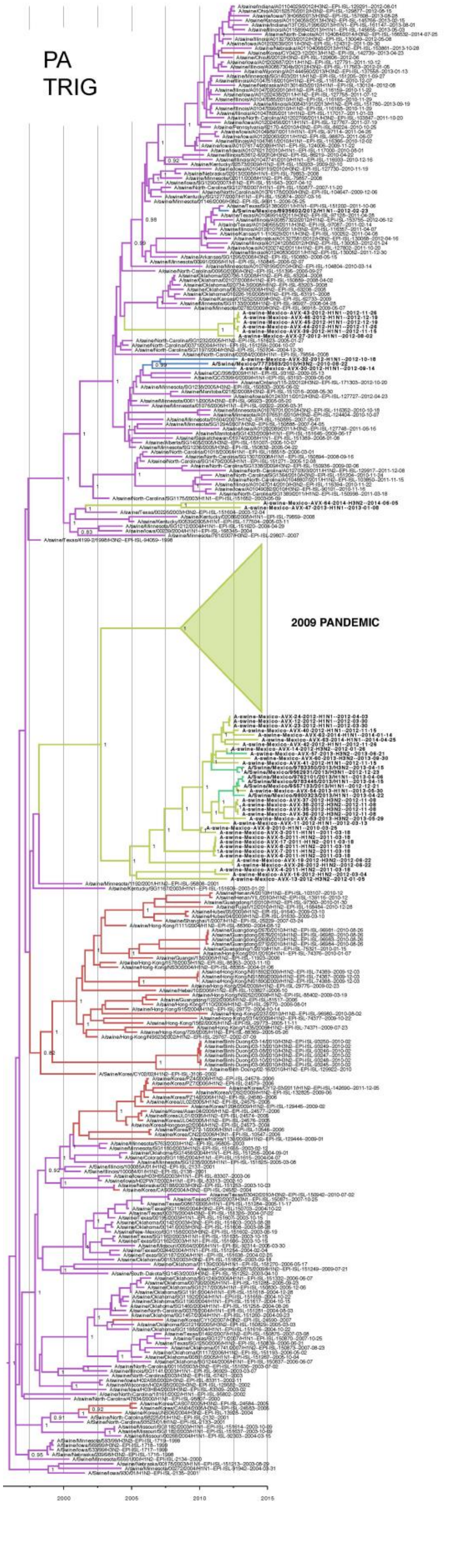
PA
TRIG
2009 PANDEMIC
2000
2005
2010
2015

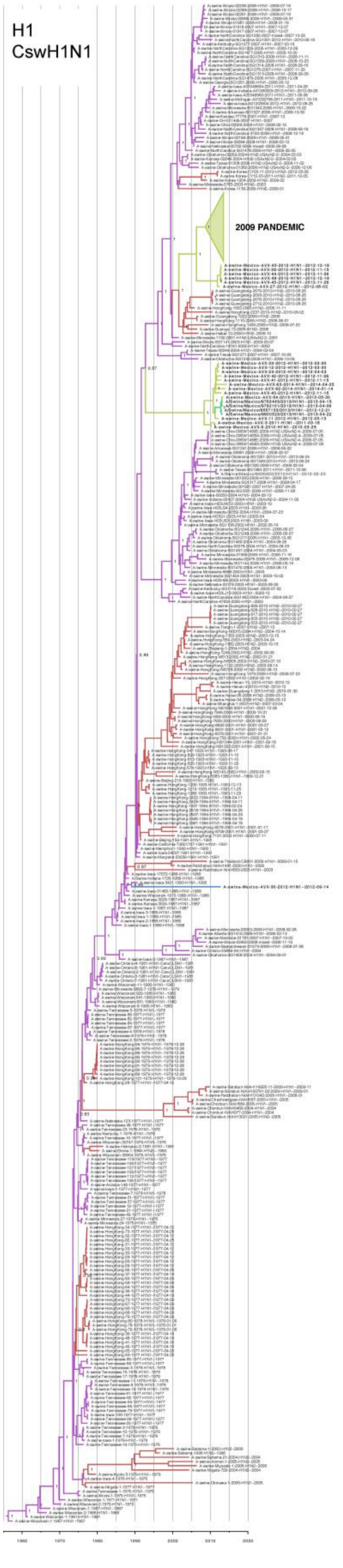
H1
CswH1N1
2009 PANDEMIC

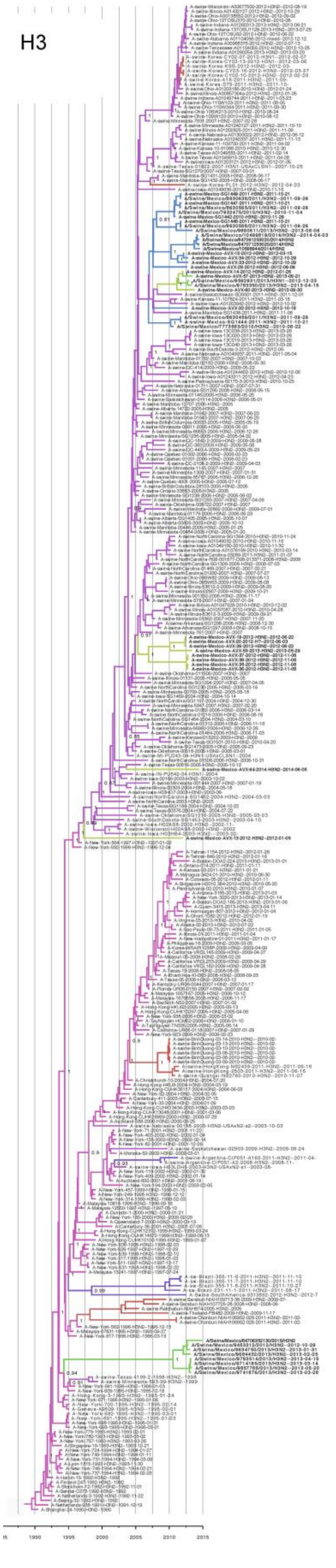
H3
1990
1995
2000
2005
2010
2015

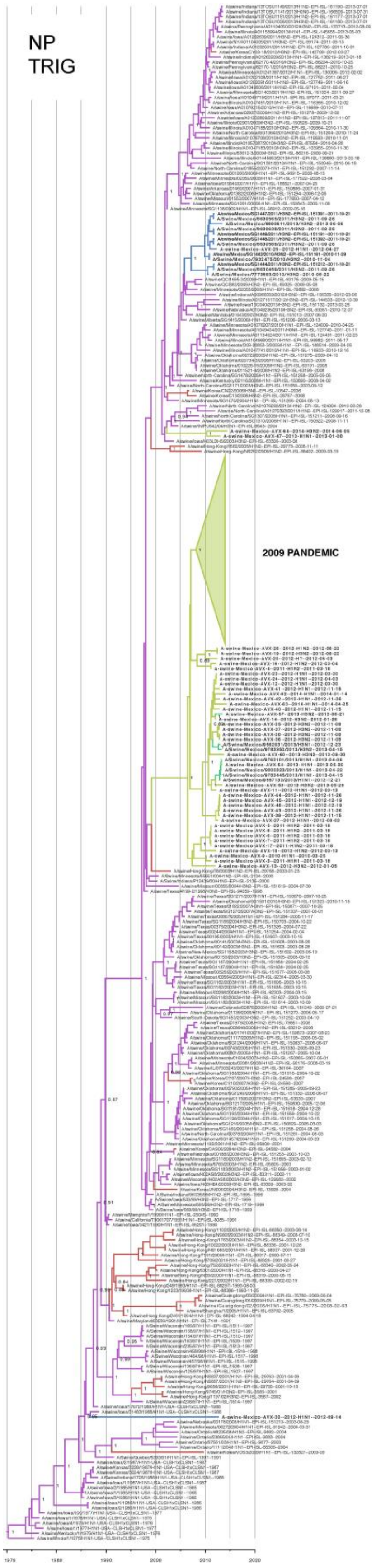
NP
TRIG
2009 PANDEMIC
1970
1980
1990
2000
2010
2020

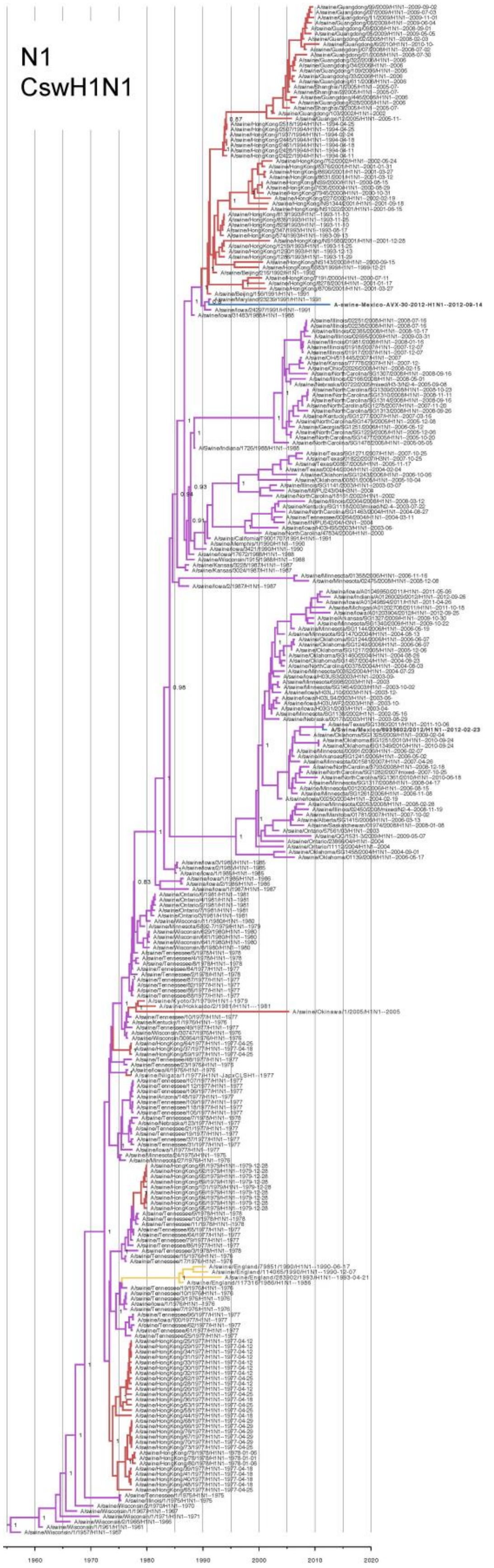
N1
CswH1N1
1960
1970
1980
1990
2000
2010
2020

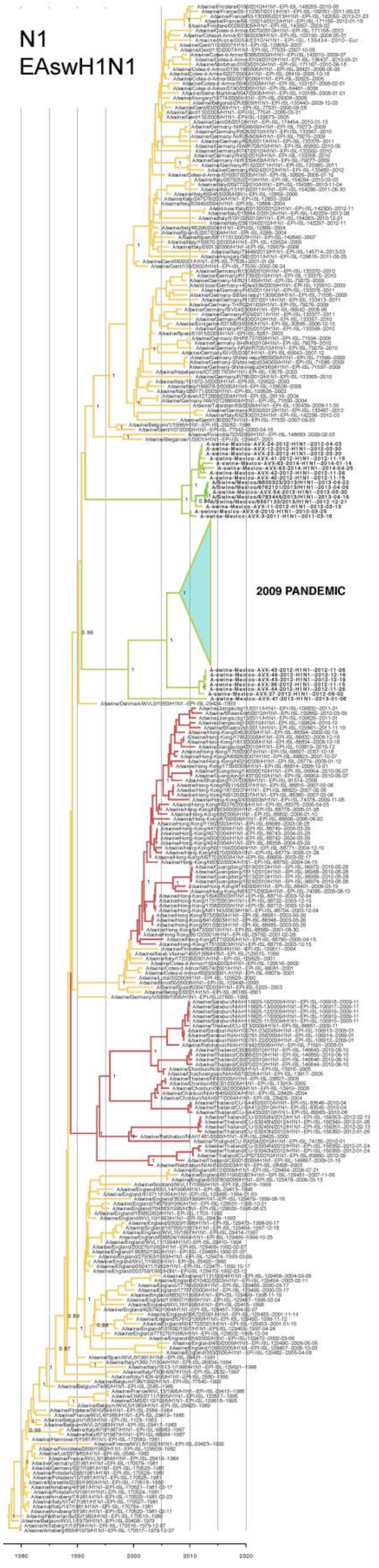
N1
EAswH1N1
2009 PANDEMIC
1980
1990
2000
2010
2020

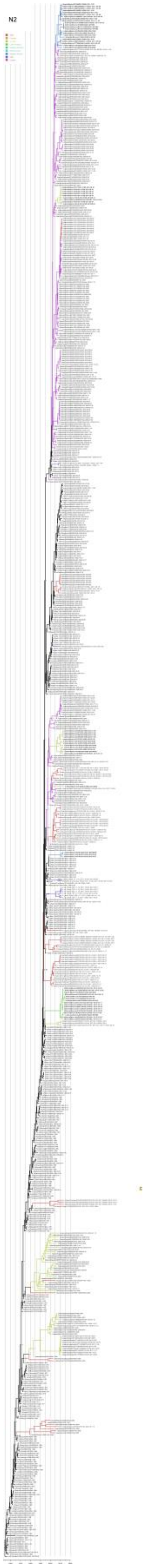
N2

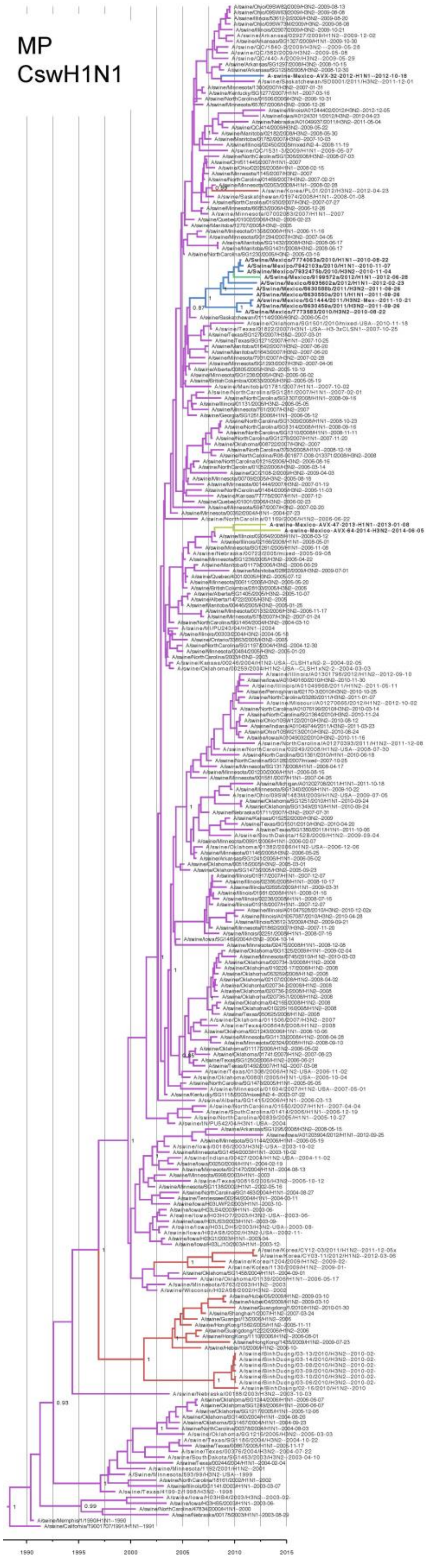
MP
CswH1N1
1990
1995
2000
2005
2010
2015

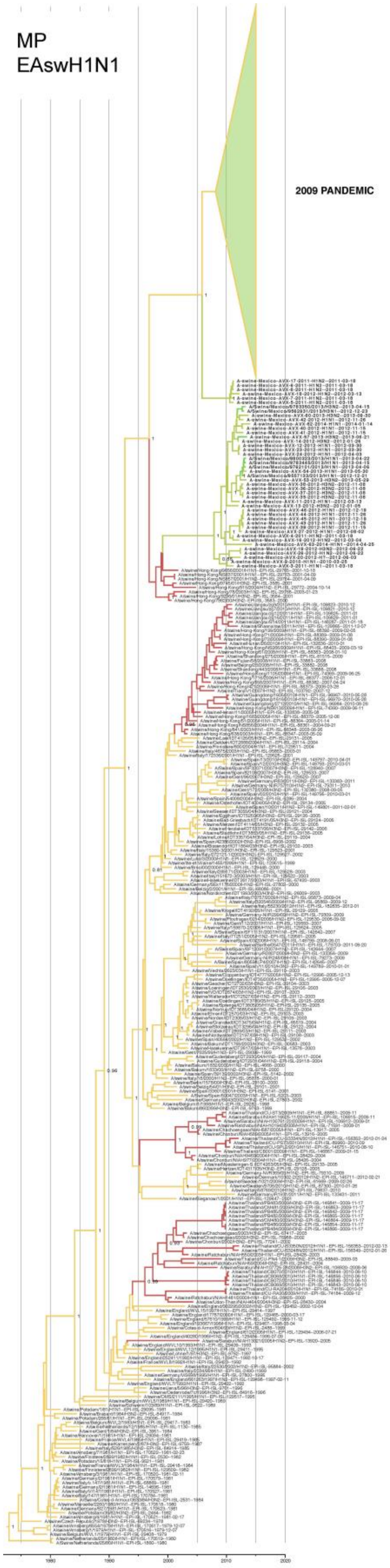
MP
EAswH1N1
2009 PANDEMIC
1980
1990
2000
2010
2020

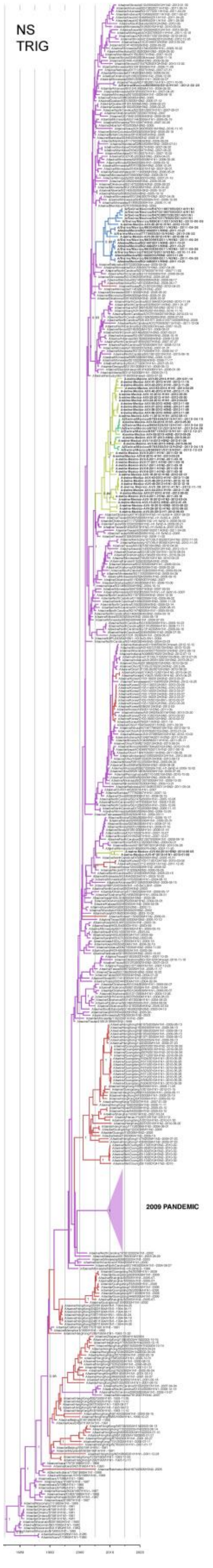
NS
TRIG
2009 PANDEMIC

Appendix Figure. Maximum clade credibility (MCC) trees inferred separately for each segment of invasive influenza virus genomes and lineages, Mexico. Time-scaled Bayesian MCC trees inferred for 13-nt alignments; PB2 (TRIG), PB1 (pdm), PB1 (TRIG), PA (TRIG), H1 (C), H3 (TR) + H3 (Hu), NP (TRIG), N1 (C), N1 (EA), N2 (TR) + N2 (Hu), MPe, MP (TRIG), and NS (TRIG). Color of each branch indicates the most probable location state. Tip labels and posterior probabilities for key nodes are provided. Numbers along branches are bootstrap values. Bold indicates strains isolated in this study. intro, introduction; H, hemagglutinin; Hu, human; MP, matrix protein; N, neuraminidase; NP, nucleoprotein; NS, nonstructural; PA, polymerase acidic; PB, polymerase basic.
